# Proteomic profiling of lung diffusion impairment in the recovery stage of SARS‐CoV‐2–induced ARDS

**DOI:** 10.1002/ctm2.838

**Published:** 2022-05-11

**Authors:** María C. García‐Hidalgo, Jessica González, Iván D. Benítez, Paola Carmona, Sally Santisteve, Anna Moncusí‐Moix, Clara Gort‐Paniello, Fátima Rodríguez‐Jara, Marta Molinero, Manel Perez‐Pons, Gerard Torres, Jesús Caballero, Carme Barberà, Ana P. Tedim, Raquel Almansa, Adrián Ceccato, Laia Fernández‐Barat, Ricard Ferrer, Dario Garcia‐Gasulla, Rosario Menéndez, Ana Motos, Oscar Peñuelas, Jordi Riera, Jesús F. Bermejo‐Martin, Antoni Torres, Ferran Barbé, David de Gonzalo‐Calvo, CIBERESUCICOVID Project (COV20/00110, ISCIII)

**Affiliations:** ^1^ Translational Research in Respiratory Medicine University Hospital Arnau de Vilanova and Santa Maria IRBLleida Lleida Spain; ^2^ CIBER of Respiratory Diseases (CIBERES) Institute of Health Carlos III Madrid Spain; ^3^ Intensive Care Department University Hospital Arnau de Vilanova, IRBLleida Lleida Spain; ^4^ Intensive Care Department University Hospital Santa María IRBLleida Lleida Spain; ^5^ Hospital Universitario Río Hortega de Valladolid Valladolid Spain; ^6^ Instituto de Investigación Biomédica de Salamanca (IBSAL) Salamanca Spain; ^7^ Servei de Pneumologia Hospital Clinic Universitat de Barcelona IDIBAPS Barcelona Spain; ^8^ Intensive Care Department Vall d'Hebron Hospital Universitari, SODIR Research Group Vall d'Hebron Institut de Recerca (VHIR) Spain; ^9^ Barcelona Supercomputing Center (BSC) Barcelona Spain; ^10^ Pulmonology Service University and Polytechnic Hospital La Fe Valencia Spain; ^11^ Hospital Universitario de Getafe Madrid Spain

Dear Editor,

In survivors of acute respiratory distress syndrome (ARDS) secondary to SARS‐CoV‐2 infection, lung diffusion impairment is consistently associated with a characteristic plasma proteome. The mechanistic pathways linked to the proteomic pattern provide novel evidence on multiple biological domains relevant to the postacute pulmonary sequelae.

Based on the increasing number of COVID‐19 survivors affected by pulmonary abnormalities and the limited understanding of the pathophysiology of the sequelae,^1^ we analysed the systemic proteomic determinants of lung diffusion impairment in SARS‐CoV‐2–induced ARDS survivors.

This is a substudy of a 3‐month prospective cohort study including survivors of severe COVID‐19 (*n* = 88).^2^ Patients admitted to the Hospital Universitari Arnau de Vilanova‐Santa María (Lleida, Spain) between March and August 2020 were included if they fulfilled the following criteria: aged over 18, developed ARDS during hospital stay and attended a ‘post‐COVID’ evaluation 3 months after hospital discharge. The study received approval from the medical ethics committee (CEIC/2273) and was performed in full compliance with the Declaration of Helsinki. The patients received written information about the study and signed an informed consent form. A complete pulmonary evaluation was performed as previously detailed.^2^

Blood samples were collected in EDTA tubes (BD, NJ, USA) and processed using standardised operating procedures with support by IRBLleida Biobank (B.0000682) and ‘Plataforma Biobancos PT20/00021’. Plasma proteomic profiling was performed using the PEA technology (Olink, Uppsala, Sweden). Four panels were analysed: organ damage, immune response, inflammation and metabolism. Additional details can be consulted at https://www.olink.com/resources‐support/document‐download‐center/. A total of 364 proteins were measured. One hundred forty‐five proteins were excluded from subsequent studies due to undetectable levels in more than 50% of the samples (Table S1). SARS‐CoV‐2 RNA was detected as previously described.^3^ STRING,^4^ Reactome,^5^ GTEX (https://www.gtexportal.org/home/) and Drug–Gene Interaction^6^ databases were used for bioinformatic analyses. All statistical analyses were performed using R software, version 4.0.2.

The study flowchart is displayed in Figure S1. The most relevant demographic and clinical characteristics during the acute phase are shown in Table 1. The median (*P*_25_;*P*_75_) age was 60.0 years (53.0;65.5), and the prevalent sex was male (69.0%). At the 3‐month follow‐up, 30% of patients presented moderate‐to‐severe pulmonary diffusion impairment (*D*_LCO _< 60%) (Table 2). Using linear models for arrays, we found 15 differentially detected proteins (FDR < 0.05) in this study group (Figure 1A, Table S2). The 15 proteins separated the patients according to the grade of lung dysfunction (Figure 1B,C). All proteins showed higher concentrations in patients with *D*_LCO _< 60% (Figure 1D). Proteins showed a dose–response relationship with *D*_LCO_ in unadjusted generalized additive models (GAM) models (Figure S2). Renal function at follow‐up was associated with both diffusion impairment and several proteins (rho≥0.3) (Table 2, Figure S3). Therefore, glomerular filtration was considered a confounder, together with age, sex, previous chronic pulmonary disease, smoking history and the use of corticoids after hospital discharge. No impact of these confounding factors was observed (Figure 1E). Except for KIM1 (rho≥0.3 and *r*_pb_≥0.3), there was no correlation between protein levels and disease severity (Figure S4). KIM1, LAMP3 and PGF correlated with the presence of fibrotic lesions (*r*_pb_≥0.3) (Figure S5). Specific correlations were observed between protein levels and laboratory parameters (rho≥0.3) (Figure S3).

**TABLE 1:** Characteristics of study sample

	All	*D*_LCO_ ≥ 60% predicted	*D*_LCO_ < 60% predicted		
	*N* = 87	*N* = 61	*N* = 26	*p*‐value	*N*
*Sociodemographic characteristics*					
Age (years)	60.0 [53.0;65.5]	56.0 [50.0;63.0]	63.5 [60.0;68.8]	0.009	87
Sex				0.193	87
Male	60 (69.0%)	39 (63.9%)	21 (80.8%)		
Female	27 (31.0%)	22 (36.1%)	5 (19.2%)		
BMI (kg/m^2^)	29.2 [25.6;33.1]	28.7 [25.8;33.2]	29.2 [25.1;31.8]	0.781	87
Smoking history				0.403	87
Former	45 (51.7%)	29 (47.5%)	16 (61.5%)		
Non‐smoker	36 (41.4%)	28 (45.9%)	8 (30.8%)		
Current	6 (6.90%)	4 (6.56%)	2 (7.69%)		
*Clinical characteristics*					
Hypertension	37 (42.5%)	22 (36.1%)	15 (57.7%)	0.103	87
Type II Diabetes Mellitus	15 (17.2%)	11 (18.0%)	4 (15.4%)	1.000	87
Obesity	36 (41.4%)	25 (41.0%)	11 (42.3%)	1.000	87
Cardiovascular disease	7 (8.05%)	3 (4.92%)	4 (15.4%)	0.190	87
Chronic lung disease	7 (8.05%)	5 (8.20%)	2 (7.69%)	1.000	87
Asthma	7 (8.05%)	5 (8.20%)	2 (7.69%)	1.000	87
Chronic kidney disease	1 (1.15%)	1 (1.64%)	0 (0.00%)	1.000	87
Chronic liver disease	3 (3.45%)	2 (3.28%)	1 (3.85%)	1.000	87
*Baseline characteristics in hospital admission*					
Time since first symptoms to hospital admission (days)	7.00 [5.00;8.00]	7.00 [5.00;8.00]	7.00 [7.00;8.00]	0.022	87
Oxygen saturation (%)	92.0 [90.0;94.0]	92.0 [90.0;95.0]	92.0 [89.0;93.0]	0.133	79
FiO_2_ (%)	28.0 [21.0;44.5]	28.0 [21.0;44.0]	24.5 [21.0;47.5]	0.937	87
PaO_2_ (mmHg)	62.0 [50.8;73.2]	64.0 [51.0;74.5]	62.0 [49.0;72.0]	0.716	80
PaCO_2_ (mmHg)	34.0 [31.0;38.0]	33.5 [30.2;37.0]	35.0 [31.0;39.0]	0.200	79
PaO_2_/FiO_2_ (mmHg)	229 [155;285]	233 [156;271]	214 [155;286]	0.697	80
Glucose (mg/dL)	120 [108;146]	119 [107;144]	120 [109;147]	0.559	87
Creatinine (mg/dL)	0.82 [0.70;0.96]	0.81 [0.67;0.94]	0.86 [0.73;0.99]	0.393	87
C‐reactive protein (mg/L)	107 [65.3;172]	107 [67.3;173]	114 [28.6;165]	0.445	84
Leukocyte count (x10^9^/L)	6.24 [5.06;9.38]	6.24 [5.05;9.06]	6.26 [5.24;9.48]	0.857	87
Neutrophil count (x10^9^/L)	5.08 [3.72;7.56]	5.08 [3.68;7.54]	4.98 [3.93;7.41]	0.893	87
Lymphocyte count (x10^9^/L)	0.83 [0.64;1.08]	0.86 [0.68;1.09]	0.80 [0.57;1.03]	0.266	87
Monocyte count (x10^9^/L)	0.32 [0.23;0.47]	0.31 [0.23;0.47]	0.35 [0.21;0.60]	0.806	87
Platelet count (x10^9^/L)	193 [152;252]	197 [161;251]	178 [143;258]	0.704	87
Urea (mg/dL)	33.0 [28.0;44.5]	31.0 [26.0;41.0]	40.0 [31.8;57.2]	0.006	87
Hospital stay					
Worst PaO_2_/FiO_2_ (mmHg)	134 [94.5;188]	134 [95.0;181]	132 [95.2;189]	1.000	87
PaO_2_/FiO_2_ categories				0.790	87
PaO_2_/FiO_2_ 201–300 mmHg	20 (23.0%)	15 (24.6%)	5 (19.2%)		
PaO_2_/FiO_2_ 101–200 mmHg	39 (44.8%)	26 (42.6%)	13 (50.0%)		
PaO_2_/FiO_2_ ≤100 mmHg	28 (32.2%)	20 (32.8%)	8 (30.8%)		
Hospital stay (days)	21.0 [12.5;35.5]	18.0 [11.0;35.0]	26.5 [14.0;36.2]	0.288	87
ICU admission	75 (86.2%)	53 (86.9%)	22 (84.6%)	0.746	87
ICU stay (days)	13.0 [5.00;25.5]	13.0 [5.00;24.0]	14.0 [6.25;30.0]	0.474	75
High‐flow nasal cannula	47 (54.0%)	34 (55.7%)	13 (50.0%)	0.798	87
Invasive mechanical ventilation	43 (49.4%)	29 (47.5%)	14 (53.8%)	0.761	87
Invasive mechanical ventilation duration (days)	17.0 [11.0;24.0]	16.0 [11.0;21.0]	18.0 [11.5;25.8]	0.467	43
Non‐invasive mechanical ventilation	45 (51.7%)	30 (49.2%)	15 (57.7%)	0.622	87
Non‐invasive mechanical ventilation duration (days)	2.50 [1.75;4.00]	2.00 [1.00;4.00]	3.00 [2.00;7.00]	0.216	44
Prone positioning	41 (47.1%)	26 (42.6%)	15 (57.7%)	0.292	87
Prone positioning duration (hours)	34.5 [19.2;62.8]	40.0 [24.0;72.0]	28.0 [8.50;47.0]	0.124	40
Antibiotics	80 (92.0%)	59 (96.7%)	21 (80.8%)	0.023	87
Hydroxychloroquine	66 (75.9%)	47 (77.0%)	19 (73.1%)	0.902	87
Tocilizumab	34 (39.1%)	25 (41.0%)	9 (34.6%)	0.751	87
Corticoids	66 (75.9%)	46 (75.4%)	20 (76.9%)	1.000	87
Remdesivir	13 (14.9%)	9 (14.8%)	4 (15.4%)	1.000	87
Lopinavir/ritonavir	64 (73.6%)	45 (73.8%)	19 (73.1%)	1.000	87
Corticoids at hospital discharge	12 (14.6%)	9 (15.5%)	3 (12.5%)	1.000	82

*Abbreviations*: BMI: body mass index; *D*_LCO_: carbon monoxide diffusing capacity; FiO_2_: fraction of inspired oxygen; ICU: intensive care unit; IMV: invasive mechanical ventilation; PaCO_2_: carbon dioxide partial pressure; PaO_2_: oxygen partial pressure; SaO_2_: arterial oxygen saturation.

^a^ Continuous variables are expressed as median [P25;P75].

^b^ Categorical variables are expressed as *n* (%).

**TABLE 2:** Pulmonary evaluation and laboratory tests at the 3‐month follow‐up

	All	*D*_LCO_ ≥ 60% predicted	*D*_LCO_ < 60% predicted		
	*N* = 87	*N* = 61	*N* = 26	*p*‐value	*N*
*Pulmonary function*					
*D*_LCO_ (% predicted)	66.1 [57.1;73.9]	71.4 [65.6;77.0]	52.8 [47.1;55.9]	<0.001	87
*D*_LCO_				<0.001	87
< 60% predicted	26 (29.9%)	0 (0.00%)	26 (100%)		
< 80% predicted	50 (57.5%)	50 (82.0%)	0 (0.00%)		
≥80% predicted	11 (12.6%)	11 (18.0%)	0 (0.00%)		
*6‐min walking test*					
Distance (m)	400 [365;430]	410 [390;448]	378 [305;404]	0.001	84
Oxygen saturation average (%)	96.0 [94.0;97.0]	96.0 [95.0;97.0]	95.0 [94.0;96.0]	0.009	85
Oxygen saturation initial (%)	97.0 [96.0;97.0]	97.0 [96.0;97.0]	96.0 [95.2;97.0]	0.005	85
Oxygen saturation final (%)	96.0 [95.0;97.0]	96.0 [95.0;97.0]	96.0 [94.0;96.0]	0.003	85
Oxygen saturation minimal (%)	95.0 [93.0;96.0]	95.0 [93.0;96.0]	94.0 [92.0;95.0]	0.017	85
*Chest CT*					
Lesions					
Reticular	42 (48.8%)	29 (48.3%)	13 (50.0%)	1.000	86
Fibrotic	20 (23.3%)	10 (16.7%)	10 (38.5%)	0.055	86
TSS score	5.00 [2.00;7.00]	2.50 [1.00;7.00]	8.00 [5.00;11.8]	<0.001	86
*Laboratory tests*					
Creatinine (mg/dl)	0.82 [0.72;0.95]	0.81 [0.71;0.91]	0.90 [0.74;1.03]	0.126	87
Glomerular filtrate (ml/min/1.73m^2^)	90.0 [83.3;90.0]	90.0 [89.4;90.0]	88.2 [74.7;90.0]	0.011	87
C‐reactive protein (mg/L)	2.40 [2.00;4.55]	2.50 [2.00;4.70]	2.20 [2.00;4.20]	0.602	87
Leucocyte count (x10^9^/l)	6.49 [5.60;7.56]	6.44 [5.73;7.36]	7.11 [5.44;8.35]	0.756	87
Neutrophil count (x10^9^/L)	3.40 [2.60;4.00]	3.53 [2.59;3.94]	3.22 [2.66;4.67]	0.770	87
Lymphocyte count (x10^9^/L)	2.12 [1.79;2.79]	2.26 [1.81;2.76]	2.08 [1.71;2.88]	0.610	87
Monocyte count (x10^9^/L)	0.54 [0.45;0.68]	0.51 [0.45;0.65]	0.59 [0.48;0.70]	0.161	87
Platelet count (x10^9^/L)	241 [210;277]	243 [217;277]	240 [197;272]	0.433	87
Urea (mg/dL)	38.0 [33.0;45.0]	37.0 [33.0;44.0]	39.0 [35.0;49.5]	0.188	87

*Abbreviations*: *D*_LCO_: carbon monoxide diffusing capacity; TSS: total severity score.

^a^ Continuous variables are expressed as median [P25;P75].

^b^ Categorical variables are expressed as n (%).

**FIGURE 1:**
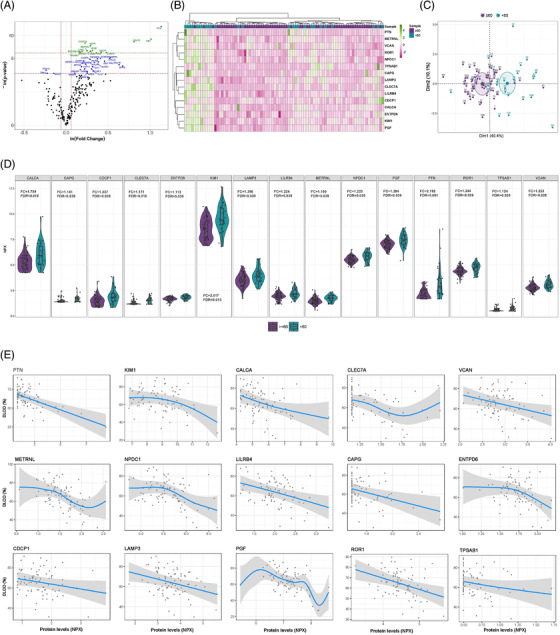
Differentially detected proteins according to the severity of lung diffusion impairment in survivors of ARDS secondary to SARS‐CoV‐2 infection. (A) Volcano plot showing the *p*‐value versus the fold change for each detected protein. Blue dots indicate significantly detected proteins considering a *p*‐value < 0.05. Green dots reflect significantly detected proteins with an FDR < 0.05. The FDR was obtained using the Benjamini–Hochberg method. (B) Heatmap representing unsupervised hierarchical clustering. Each column represents a survivor. Each row represents a differentially detected protein. The patient clustering tree is plotted on top. The protein clustering is shown on the left. Protein levels are represented through a colour scale, with green tones related to increasing levels and pink tones related to decreasing expression. (C) Principal component analysis using differentially detected proteins. Each point denotes a survivor and is represented with a specific colour depending on the presence or absence of severe diffusion impairment. (D) Violin plots of differentially detected proteins. Fold change (FC) and FDR are plotted for each protein. (E) Generalised additive model (GAM) with penalized cubic regression splines for *D*_LCO_ (Y axis) and the levels of each of the differentially detected proteins (NPX) (X axis). The association was adjusted by age, sex, previous chronic pulmonary disease, smoking history, the use of corticoids after hospital discharge and glomerular filtration. All proteins included in the analysis showed an FDR < 0.05

The sparse partial least‐squares discriminant analysis (sPLS‐DA) generated a signature of 20 proteins that allowed optimal discrimination between study groups (AUC = 0.872) (Figure 2A–C). Based on the variable importance of component 1, the top five relevant contributors were PTN, KIM1, CALCA, CLEC7A and ENTPD6 (Figure 2A). The feature selection procedure based on random forest supported these results (Figure S6A). In addition, sPLS was used to determine the protein profile that best explained the *D*_LCO_ levels (as a continuous variable) (Figures 2D,E,F). The analysis identified a signature of 35 proteins. PTN, PGF, NPDC1 and METRNL were the most weighted factors for defining component 1 (Figure 2D). The proteomic profile generated using random forest was in concordance with these findings (Figure S6B). IFN‐γ, which participates in the response to infection,^7^ was associated with diffusion capacity. Therefore, we analysed viral load in plasma samples from a subset of 50 patients. Only one patient was positive for the presence of SARS‐CoV‐2 RNA.

**FIGURE 2:**
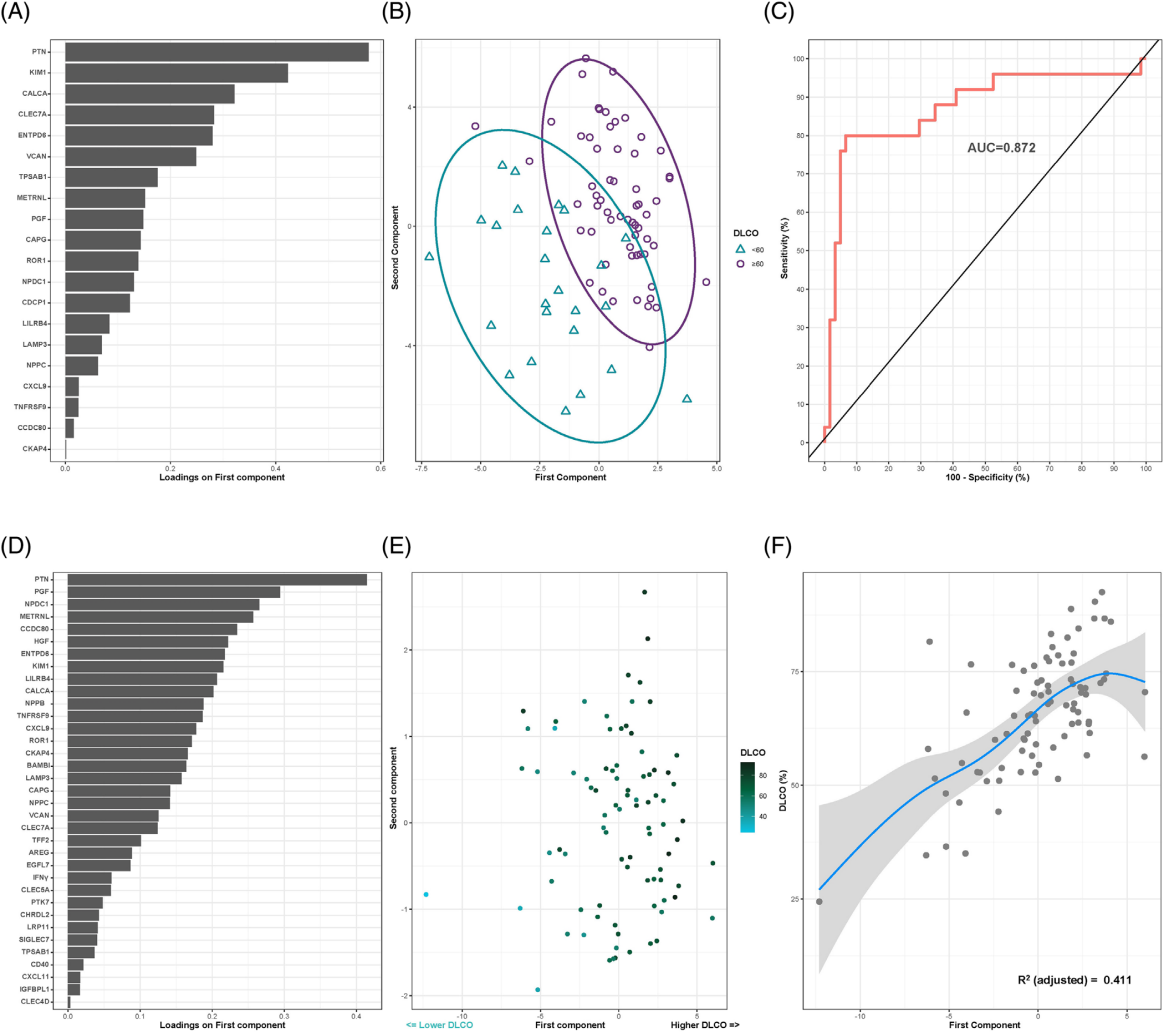
Plasma proteomic signatures associated with moderate/severe diffusion impairment and *D*_LCO_ levels in survivors of ARDS secondary to SARS‐CoV‐2 infection. (A) Proteins are ranked by their variable importance to component 1. (B) Supervised component analysis cluster through sparse partial least‐squares discriminant analysis (sPLS‐DA) discriminating between survivors with moderate/severe diffusion impairment (D_LCO _< 60%) and survivors with mild or an absence of alterations in diffusion capacity survivors (D_LCO_≥60%). Each point represents a patient. (C) Receiver operating characteristic (ROC) curve for the protein signature. The discriminative power of the signature is represented as the area under the ROC curve (AUC). (D) Proteins ranked by their variable importance for component 1. (E) Supervised component analysis cluster through sPLS according to the *D*_LCO_ levels of the cohort. Each protein represents a survivor. (F) Generalized additive model (GAM) with penalized cubic regression splines for *D*_LCO_ (*Y* axis) and the first component (*X* axis). The significance of the association is given by the coefficient of determination (*R* ^2^)

The signature including the higher number of proteins (*n* = 35) was used for bioinformatic analyses. An enrichment in pathways associated with cell proliferation and differentiation, tissue remodelling, inflammation and immune response, angiogenesis, coagulation and fibrosis was observed (Figure S7A, Tables S3 and S4). Three independent protein networks were identified (Figure S8). A generalized expression of the signature was observed in the lung but also in other tissues (Figure S7B). The proteomic pattern was enriched in lung epithelial, endothelial and immune cells (Figure S7C). The drug–gene interaction analysis identified several FDA‐approved drugs that can target the proteins (Table S5).

Postinfection long‐term lung dysfunction has become clinically evident in a large percentage of SARS‐CoV‐2–induced‐ARDS survivors. Systemic molecular profiling constitutes a promising strategy to decipher the underlying biological mechanisms linked to the pulmonary outcomes and, consequently, to identify candidates that may be amenable of therapeutic intervention.^8^, ^9^, ^10^ Here, we provide compelling evidence that (i) a set of plasma proteins are differentially detected in survivors with moderate‐to‐severe diffusion impairment; (ii) diffusion capacity is associated with alterations in the proteomic profile, even after adjustment for confounding factors; (iii) survivors with the most serious sequelae show higher disturbances in the protein levels; (iv) sPLS and random forest define protein signatures highly associated with pulmonary function; (v) the signatures are composed of heterogeneous factors implicating multiple biological pathways; (vi) the signatures constitute a source of targets for candidate drugs; (vii) plasma proteomic profiles accurately classify patients with respiratory sequelae; and (viii) no association was observed between blood viral load and diffusion impairment.

## CONCLUSION

The plasma proteomic profile linked to lung diffusion impairment improves our understanding of the physiopathology of postacute pulmonary sequelae in COVID‐19, and, consequently, constitutes a useful resource for the design of therapeutic strategies and the development of tools to improve medical decision‐making in the “post‐COVID” syndrome. Additional cohorts and functional analyses are needed to corroborate our findings.

## CONFLICT OF INTEREST

The authors declare that they have no competing interests.

## FUNDING INFORMATION

Financial support was provided by the Instituto de Salud Carlos III de Madrid (COV20/00110), co‐funded by the European Development Regional Fund (A Way to Achieve Europe programme) and Centro de Investigación Biomedica En Red Enfermedades Respiratorias (CIBERES). CIBERES is an initiative of the Instituto de Salud Carlos III. Suported by: Programa de donaciones “estar preparados” UNESPA (Madrid, Spain); and Fundación Francisco Soria Melguizo (Madrid, Spain). Finançat per La Fundació La Marató de TV3, projecte amb codi 202108‐30/‐31. COVIDPONENT is funded by Institut Català de la Salut and Gestió de Serveis Sanitaris. APT was funded by the Sara Borrell Research Grant CD018/0123 funded by the Instituto de Salud Carlos III and co‐financed by the European Development Regional Fund (A Way to Achieve Europe programme). MCGH is the recipient of a predoctoral fellowship from the “University of Lleida”. DdGC (Miguel Servet 2020: CP20/00041) and MM (PFIS: FI21/00187) have received financial support from the Instituto de Salud Carlos III, cofunded by the European Social Fund (ESF)/“Investing in your future”.

## Supporting information



Supporting Material
