# Supplementary material for: Proteomic profiling of lung diffusion impairment in the recovery stage of SARS‐CoV‐2–induced ARDS
Source: Clin Transl Med. 2022 May 11;12(5):e838. doi: 10.1002/ctm2.838 (PMC9091985; doi:10.1002/ctm2.838)
Supplement: Supplementary file 1 — Supporting Material [file CTM2-12-0-s001.docx]

**Online data supplement**

**Proteomic profiling of lung diffusion impairment in the recovery stage of SARS-CoV-2–induced ARDS**

María C. García-Hidalgo,^1^ Jessica González,^1,2^ Iván D. Benítez,^1,2^ Paola Carmona,^1^ Sally Santisteve,^1^ Anna Moncusí-Moix,^1,2^ Clara Gort-Paniello,^1,2^ Fátima Rodríguez-Jara,^1,2^ Marta Molinero,^1^ Manel Pérez-Pons,^1,2^ Gerard Torres,^1,2^ Jesús Caballero,^3^ Carme Barberà,^4^ Ana P. Tedim,^5^ Raquel Almansa,^2,5^ Adrián Ceccato,^2^ Laia Fernández-Barat,^2,6^ Ricard Ferrer,^2,7^ Dario Garcia-Gasulla,^8^ Rosario Menéndez,^2,9^ Ana Motos,^2,6^ Oscar Peñuelas,^2,10^ Jordi Riera,^2,7^ Jesús F. Bermejo-Martin,^2,5^ Antoni Torres,^2,6^ Ferran Barbé,^1,2^ David de Gonzalo-Calvo,^1,2,*^

*on behalf of the CIBERESUCICOVID Project (COV20/00110, ISCIII).*

**SUPPLEMENTAL TABLES**

**Supplemental Table S1.** Non-detected proteins.

| Non expressed proteins | Non-detected (%) | Non expressed proteins | Non-detected (%) |
| --- | --- | --- | --- |
| IRAK4 | 100.0% | **IRF9** | 95.4% |
| DGKZ | 100.0% | **ARNT** | 95.4% |
| TRAF2 | 100.0% | **IL-20RA** | 95.4% |
| EGLN1 | 100.0% | **SIRT2** | 95.4% |
| LY75 | 100.0% | **KLK10** | 95.4% |
| CD28 | 100.0% | **TIGAR** | 95.4% |
| BIRC2 | 100.0% | **CES2** | 95.4% |
| TREM1 | 100.0% | **LTA4H** | 95.4% |
| ICA1 | 100.0% | **HPGDS** | 95.4% |
| CXCL12 | 100.0% | **PRKCQ** | 94.3% |
| CLEC6A | 100.0% | **SRPK2** | 94.3% |
| IL12RB1 | 100.0% | **DAPP1** | 94.3% |
| TANK | 100.0% | **PADI2** | 94.3% |
| GDNF | 100.0% | **IL-2RB** | 94.3% |
| IL2 | 100.0% | **IL-15RA** | 94.3% |
| Beta-NGF | 100.0% | **IL-24** | 94.3% |
| ANGPT2 | 100.0% | **IL10** | 94.3% |
| FKBP4 | 100.0% | **ST1A1** | 94.3% |
| CLSPN | 100.0% | **RASA1** | 94.3% |
| CLEC1A | 100.0% | **JUN** | 93.1% |
| PXN | 100.0% | **TSLP** | 93.1% |
| ALDH3A1 | 100.0% | **IL13** | 93.1% |
| CSNK1D | 100.0% | **CNTNAP2** | 92.0% |
| MVK | 100.0% | **IL4** | 92.0% |
| ADGRG1 | 100.0% | **PRKAB1** | 92.0% |
| NOS3 | 100.0% | **PRKRA** | 92.0% |
| EDIL3 | 100.0% | **PDCD1** | 92.0% |
| PPM1B | 100.0% | **ANGPTL1** | 90.8% |
| ENTPD2 | 100.0% | **EPO** | 89.7% |
| PDP1 | 100.0% | **PPP1R9B** | 88.5% |
| ITGB1BP1 | 100.0% | **PLXNA4** | 88.5% |
| CNTN2 | 100.0% | **SMAD1** | 87.4% |
| GALNT3 | 98.9% | **IL5** | 86.2% |
| EIF5A | 98.9% | **TNNI3** | 86.2% |
| NF2 | 98.9% | **TRIM21** | 85.1% |
| FCRL3 | 98.9% | **FGF2** | 83.9% |
| PIK3AP1 | 98.9% | **ITGB7** | 83.9% |
| SH2D1A | 98.9% | **LRP1** | 83.9% |
| IFNLR1 | 98.9% | **FGR** | 81.6% |
| DDX58 | 98.9% | **WAS** | 81.6% |
| FGF-23 | 98.9% | **VASH1** | 80.5% |
| ARTN | 98.9% | **PSIP1** | 79.3% |
| IL-20 | 98.9% | **ITM2A** | 79.3% |
| QDPR | 98.9% | **FOSB** | 79.3% |
| APEX1 | 98.9% | **TRIM5** | 78.2% |
| SIRT5 | 98.9% | **INPPL1** | 78.2% |
| FES | 98.9% | **SERPINA9** | 78.2% |
| KIR3DL1 | 98.9% | **IRAK1** | 75.9% |
| RCOR1 | 98.9% | **SH2B3** | 74.7% |
| PON2 | 98.9% | **S100P** | 74.7% |
| NUB1 | 98.9% | **IL-10RA** | 73.6% |
| STXBP3 | 98.9% | **REG4** | 73.6% |
| KPNA1 | 97.7% | **DPP6** | 72.4% |
| FGF-5 | 97.7% | **MAP4K5** | 72.4% |
| IL-22 RA1 | 97.7% | **DPP10** | 71.3% |
| IL33 | 97.7% | **NFATC3** | 69.0% |
| LIF | 97.7% | **AIFM1** | 69.0% |
| NRTN | 97.7% | **CANT1** | 67.8% |
| ANXA4 | 97.7% | **USP8** | 67.8% |
| COMT | 97.7% | **FABP9** | 67.8% |
| METAP1 | 97.7% | **CLSTN2** | 65.5% |
| ATP6AP2 | 97.7% | **LAG3** | 64.4% |
| NCF2 | 97.7% | **PRDX3** | 62.1% |
| IL-1 alpha | 96.6% | **BTC** | 60.9% |
| CA13 | 96.6% | **NBN** | 58.6% |
| CA12 | 96.6% | **MAEA** | 57.5% |
| ERBB2IP | 96.6% | **GALNT10** | 57.5% |
| MAGED1 | 96.6% | **AGR2** | 56.3% |
| RARRES1 | 96.6% | **NTF4** | 55.2% |
| RASSF2 | 96.6% | **IL-17A** | 55.2% |
| CRH | 96.6% | **MCP-3** | 54.0% |
| PSMA1 | 96.6% | **ENPP7** | 50.6% |
| RRM2B | 96.6% |  |  |

**Supplemental Table S2.** Detected proteins.

| Expressed proteins | Detected (%) | FC | FDR p-value | AUC |
| --- | --- | --- | --- | --- |
| PTN | 74.7% | 2.192 | 0.001 | 0.754 |
| KIM1 | 100.0% | 2.017 | 0.013 | 0.726 |
| CLEC7A | 54.0% | 1.171 | 0.018 | 0.714 |
| CALCA | 93.1% | 1.754 | 0.018 | 0.717 |
| VCAN | 100.0% | 1.223 | 0.028 | 0.721 |
| TPSAB1 | 52.9% | 1.124 | 0.039 | 0.699 |
| CAPG | 52.9% | 1.141 | 0.039 | 0.719 |
| CDCP1 | 92.0% | 1.337 | 0.039 | 0.683 |
| LILRB4 | 97.7% | 1.224 | 0.039 | 0.682 |
| LAMP3 | 100.0% | 1.396 | 0.039 | 0.681 |
| NPDC1 | 100.0% | 1.223 | 0.039 | 0.699 |
| METRNL | 100.0% | 1.190 | 0.039 | 0.723 |
| ROR1 | 100.0% | 1.244 | 0.039 | 0.706 |
| PGF | 100.0% | 1.284 | 0.039 | 0.696 |
| ENTPD6 | 100.0% | 1.112 | 0.039 | 0.717 |
| NPPC | 100.0% | 1.441 | 0.078 | 0.670 |
| CXCL9 | 98.9% | 1.435 | 0.084 | 0.706 |
| TNFRSF9 | 98.9% | 1.297 | 0.084 | 0.671 |
| CCDC80 | 100.0% | 1.330 | 0.084 | 0.721 |
| CKAP4 | 98.9% | 1.219 | 0.087 | 0.690 |
| CLMP | 71.3% | 1.158 | 0.105 | 0.679 |
| IFN-gamma | 80.5% | 1.373 | 0.105 | 0.638 |
| IGFBPL1 | 83.9% | 1.141 | 0.112 | 0.683 |
| BAMBI | 88.5% | 1.146 | 0.112 | 0.657 |
| PTK7 | 95.4% | 1.227 | 0.112 | 0.647 |
| HGF | 98.9% | 1.306 | 0.112 | 0.671 |
| PILRB | 98.9% | 1.263 | 0.112 | 0.672 |
| KLRD1 | 100.0% | 1.300 | 0.122 | 0.655 |
| LRP11 | 100.0% | 1.175 | 0.122 | 0.664 |
| CCL19 | 98.9% | 1.549 | 0.128 | 0.618 |
| NT-proBNP | 97.7% | 1.596 | 0.146 | 0.639 |
| SIT1 | 89.7% | 1.244 | 0.149 | 0.650 |
| NPTXR | 100.0% | 1.137 | 0.149 | 0.653 |
| CD83 | 100.0% | 1.167 | 0.188 | 0.631 |
| CLUL1 | 100.0% | 1.235 | 0.188 | 0.627 |
| SEMA3F | 100.0% | 1.125 | 0.198 | 0.634 |
| ITGA11 | 96.6% | 0.848 | 0.209 | 0.649 |
| IL18 | 98.9% | 1.251 | 0.223 | 0.684 |
| AREG | 100.0% | 1.191 | 0.227 | 0.631 |
| NQO2 | 57.5% | 0.905 | 0.253 | 0.628 |
| TRAIL | 98.9% | 0.856 | 0.253 | 0.613 |
| MASP1 | 100.0% | 1.075 | 0.253 | 0.627 |
| CHRDL2 | 100.0% | 1.191 | 0.253 | 0.617 |
| CXCL11 | 98.9% | 1.386 | 0.268 | 0.618 |
| PTH1R | 96.6% | 1.137 | 0.292 | 0.646 |
| CD79B | 100.0% | 1.116 | 0.292 | 0.637 |
| ITGB6 | 100.0% | 1.128 | 0.313 | 0.605 |
| SIGLEC7 | 100.0% | 1.092 | 0.315 | 0.596 |
| CD40 | 98.9% | 1.199 | 0.332 | 0.616 |
| CCL11 | 100.0% | 1.115 | 0.551 | 0.567 |
| CDH2 | 100.0% | 1.116 | 0.343 | 0.620 |
| IL-12B | 98.9% | 1.135 | 0.345 | 0.592 |
| CCL4 | 98.9% | 1.202 | 0.365 | 0.656 |
| DNER | 98.9% | 0.863 | 0.365 | 0.624 |
| EGFL7 | 96.6% | 1.114 | 0.385 | 0.612 |
| RNASE3 | 100.0% | 0.695 | 0.385 | 0.611 |
| SSC4D | 98.9% | 1.442 | 0.391 | 0.583 |
| CCL20 | 98.9% | 1.365 | 0.398 | 0.637 |
| LILRA5 | 100.0% | 1.102 | 0.410 | 0.583 |
| ANXA11 | 71.3% | 0.800 | 0.430 | 0.556 |
| CXCL10 | 98.9% | 1.253 | 0.430 | 0.658 |
| MCP-2 | 98.9% | 1.217 | 0.431 | 0.608 |
| TSHB | 100.0% | 0.838 | 0.435 | 0.593 |
| CCL3 | 98.9% | 1.215 | 0.446 | 0.679 |
| EN-RAGE | 98.9% | 1.098 | 0.446 | 0.603 |
| SLAMF1 | 80.5% | 1.095 | 0.450 | 0.625 |
| DPP7 | 97.7% | 1.219 | 0.450 | 0.659 |
| MCP-1 | 98.9% | 1.144 | 0.450 | 0.607 |
| CXCL6 | 98.9% | 1.178 | 0.450 | 0.621 |
| KRT19 | 100.0% | 1.231 | 0.450 | 0.604 |
| OPG | 98.9% | 1.136 | 0.470 | 0.633 |
| NUCB2 | 98.9% | 1.231 | 0.471 | 0.611 |
| BTN3A2 | 100.0% | 1.188 | 0.477 | 0.544 |
| IL-10RB | 98.9% | 1.115 | 0.491 | 0.622 |
| CLEC5A | 100.0% | 1.059 | 0.491 | 0.584 |
| NCR1 | 82.8% | 1.081 | 0.496 | 0.598 |
| CLEC4A | 63.2% | 0.953 | 0.499 | 0.585 |
| ARG1 | 60.9% | 0.852 | 0.501 | 0.517 |
| DIABLO | 62.1% | 0.872 | 0.501 | 0.524 |
| CX3CL1 | 97.7% | 1.114 | 0.501 | 0.633 |
| PD-L1 | 98.9% | 1.100 | 0.501 | 0.638 |
| TNF | 98.9% | 1.086 | 0.501 | 0.584 |
| CSF-1 | 98.9% | 1.086 | 0.501 | 0.659 |
| CLEC4D | 100.0% | 1.131 | 0.501 | 0.595 |
| STC1 | 100.0% | 1.146 | 0.501 | 0.582 |
| DFFA | 100.0% | 1.178 | 0.501 | 0.554 |
| Flt3L | 98.9% | 1.113 | 0.506 | 0.585 |
| TFF2 | 100.0% | 1.168 | 0.506 | 0.612 |
| TGF-alpha | 98.9% | 1.078 | 0.509 | 0.619 |
| SPRY2 | 94.3% | 1.263 | 0.526 | 0.528 |
| CD5 | 98.9% | 1.090 | 0.532 | 0.581 |
| CXCL1 | 98.9% | 0.866 | 0.550 | 0.574 |
| IL6 | 60.9% | 1.085 | 0.644 | 0.590 |
| CCL23 | 98.9% | 1.141 | 0.551 | 0.591 |
| GHRL | 100.0% | 0.865 | 0.551 | 0.608 |
| BACH1 | 89.7% | 1.121 | 0.562 | 0.547 |
| HCLS1 | 98.9% | 1.177 | 0.562 | 0.538 |
| NADK | 100.0% | 1.088 | 0.562 | 0.596 |
| RTN4R | 100.0% | 1.066 | 0.562 | 0.561 |
| CST5 | 98.9% | 1.128 | 0.565 | 0.595 |
| ACP6 | 98.9% | 0.911 | 0.567 | 0.558 |
| CALR | 100.0% | 1.153 | 0.589 | 0.579 |
| PRDX5 | 98.9% | 1.189 | 0.591 | 0.539 |
| ADGRE2 | 100.0% | 1.074 | 0.602 | 0.585 |
| PRDX1 | 100.0% | 1.160 | 0.605 | 0.525 |
| HEXIM1 | 100.0% | 1.192 | 0.605 | 0.535 |
| FAM3C | 100.0% | 1.115 | 0.605 | 0.573 |
| AHCY | 85.1% | 0.942 | 0.608 | 0.555 |
| ENTPD5 | 93.1% | 1.049 | 0.608 | 0.552 |
| FGF-19 | 98.9% | 0.863 | 0.608 | 0.597 |
| ALDH1A1 | 100.0% | 0.909 | 0.608 | 0.532 |
| MEP1B | 86.2% | 0.902 | 0.630 | 0.556 |
| CLEC4C | 94.3% | 0.899 | 0.639 | 0.578 |
| IL8 | 98.9% | 1.105 | 0.639 | 0.667 |
| TRANCE | 98.9% | 0.905 | 0.639 | 0.558 |
| LRIG1 | 100.0% | 1.050 | 0.639 | 0.578 |
| GAL | 100.0% | 1.122 | 0.639 | 0.568 |
| KYAT1 | 100.0% | 0.910 | 0.639 | 0.557 |
| FXYD5 | 59.8% | 1.062 | 0.644 | 0.537 |
| SERPINB8 | 69.0% | 1.062 | 0.644 | 0.625 |
| SUMF2 | 70.1% | 0.959 | 0.644 | 0.516 |
| MGMT | 92.0% | 0.895 | 0.644 | 0.552 |
| TYMP | 100.0% | 0.863 | 0.644 | 0.559 |
| ENAH | 56.3% | 1.038 | 0.647 | 0.566 |
| MAX | 72.4% | 1.092 | 0.647 | 0.545 |
| HNMT | 100.0% | 1.093 | 0.647 | 0.557 |
| NT-3 | 52.9% | 1.064 | 0.673 | 0.507 |
| EIF4G1 | 86.2% | 0.955 | 0.673 | 0.527 |
| AMN | 95.4% | 1.100 | 0.673 | 0.607 |
| PAG1 | 100.0% | 0.866 | 0.673 | 0.504 |
| TMPRSS15 | 63.2% | 0.946 | 0.696 | 0.547 |
| CD8A | 98.9% | 1.100 | 0.698 | 0.530 |
| CA14 | 100.0% | 1.078 | 0.709 | 0.559 |
| DCBLD2 | 100.0% | 1.040 | 0.734 | 0.539 |
| IL-17C | 90.8% | 1.080 | 0.739 | 0.542 |
| MMP-1 | 98.9% | 1.184 | 0.739 | 0.544 |
| TINAGL1 | 100.0% | 1.034 | 0.739 | 0.550 |
| FGF-21 | 97.7% | 1.124 | 0.741 | 0.540 |
| SCF | 98.9% | 0.935 | 0.741 | 0.535 |
| TNFB | 98.9% | 0.949 | 0.741 | 0.546 |
| GRAP2 | 98.9% | 0.856 | 0.743 | 0.520 |
| CDSN | 98.9% | 1.084 | 0.745 | 0.526 |
| ZBTB16 | 62.1% | 1.111 | 0.751 | 0.500 |
| FOXO1 | 66.7% | 1.050 | 0.751 | 0.578 |
| ADA | 98.9% | 0.946 | 0.751 | 0.510 |
| TYRO3 | 100.0% | 1.026 | 0.751 | 0.552 |
| FBP1 | 100.0% | 0.886 | 0.751 | 0.537 |
| THOP1 | 88.5% | 0.963 | 0.771 | 0.516 |
| FCRL6 | 96.6% | 1.044 | 0.771 | 0.541 |
| ANGPTL7 | 96.6% | 1.026 | 0.771 | 0.515 |
| CD244 | 98.9% | 1.051 | 0.771 | 0.563 |
| uPA | 98.9% | 1.043 | 0.771 | 0.551 |
| OSM | 98.9% | 1.075 | 0.771 | 0.571 |
| BAG6 | 100.0% | 0.957 | 0.779 | 0.512 |
| CLEC4G | 96.6% | 1.034 | 0.800 | 0.559 |
| TOP2B | 56.3% | 1.084 | 0.804 | 0.585 |
| CTSO | 100.0% | 1.021 | 0.815 | 0.554 |
| CD2AP | 100.0% | 0.888 | 0.819 | 0.533 |
| GLRX | 97.7% | 0.961 | 0.825 | 0.501 |
| PTPRJ | 51.7% | 0.946 | 0.831 | 0.521 |
| FAM3B | 54.0% | 0.980 | 0.831 | 0.503 |
| ENO2 | 71.3% | 0.967 | 0.831 | 0.538 |
| YES1 | 97.7% | 1.073 | 0.831 | 0.546 |
| PLIN1 | 98.9% | 0.968 | 0.831 | 0.536 |
| MILR1 | 100.0% | 1.044 | 0.831 | 0.526 |
| SOST | 100.0% | 1.050 | 0.831 | 0.555 |
| CRKL | 100.0% | 0.923 | 0.831 | 0.512 |
| ST3GAL1 | 100.0% | 1.041 | 0.831 | 0.595 |
| DAB2 | 93.1% | 0.900 | 0.850 | 0.520 |
| CASP-8 | 97.7% | 0.969 | 0.850 | 0.515 |
| STAMBP | 98.9% | 0.965 | 0.858 | 0.506 |
| HDGF | 59.8% | 0.968 | 0.864 | 0.522 |
| ITGA6 | 77.0% | 1.029 | 0.864 | 0.577 |
| DSG4 | 97.7% | 0.967 | 0.864 | 0.512 |
| STX8 | 100.0% | 1.049 | 0.864 | 0.538 |
| HSD11B1 | 100.0% | 0.972 | 0.870 | 0.515 |
| LHB | 52.9% | 0.968 | 0.874 | 0.511 |
| EDAR | 85.1% | 1.035 | 0.874 | 0.523 |
| DCTN1 | 87.4% | 0.974 | 0.874 | 0.516 |
| FCRL1 | 90.8% | 1.015 | 0.874 | 0.520 |
| GLB1 | 98.9% | 1.036 | 0.874 | 0.538 |
| VEGFA | 98.9% | 1.025 | 0.874 | 0.560 |
| CD6 | 98.9% | 0.967 | 0.874 | 0.505 |
| MCP-4 | 98.9% | 1.043 | 0.874 | 0.539 |
| TNFSF14 | 98.9% | 1.032 | 0.874 | 0.536 |
| LIF-R | 98.9% | 0.983 | 0.874 | 0.500 |
| IL-18R1 | 98.9% | 1.026 | 0.874 | 0.556 |
| CXCL5 | 98.9% | 0.957 | 0.874 | 0.501 |
| MMP-10 | 98.9% | 1.023 | 0.874 | 0.548 |
| 4E-BP1 | 98.9% | 0.970 | 0.874 | 0.504 |
| TWEAK | 98.9% | 1.023 | 0.874 | 0.523 |
| CD164 | 100.0% | 0.974 | 0.874 | 0.501 |
| DDC | 100.0% | 0.980 | 0.874 | 0.513 |
| ADGRG2 | 100.0% | 1.012 | 0.874 | 0.513 |
| SNAP23 | 100.0% | 0.927 | 0.874 | 0.532 |
| NOMO1 | 100.0% | 1.014 | 0.874 | 0.513 |
| TXNDC5 | 100.0% | 1.036 | 0.874 | 0.526 |
| PPP1R2 | 100.0% | 0.943 | 0.874 | 0.517 |
| MCFD2 | 100.0% | 1.025 | 0.874 | 0.525 |
| CD1C | 100.0% | 1.021 | 0.874 | 0.522 |
| SDC4 | 100.0% | 0.969 | 0.874 | 0.516 |
| PDGFC | 100.0% | 1.011 | 0.874 | 0.558 |
| PLXDC1 | 100.0% | 0.984 | 0.874 | 0.532 |
| PVALB | 100.0% | 0.939 | 0.874 | 0.538 |
| BANK1 | 100.0% | 1.028 | 0.887 | 0.524 |
| IL7 | 98.9% | 0.985 | 0.892 | 0.506 |
| AXIN1 | 98.9% | 0.976 | 0.892 | 0.504 |
| CCL28 | 98.9% | 1.009 | 0.915 | 0.523 |
| NECTIN2 | 100.0% | 1.013 | 0.915 | 0.543 |
| SERPINB6 | 100.0% | 0.984 | 0.917 | 0.506 |
| LAP TGF-beta-1 | 98.9% | 1.014 | 0.957 | 0.512 |
| FKBP1B | 98.9% | 1.013 | 0.957 | 0.509 |
| CDHR5 | 100.0% | 1.005 | 0.961 | 0.506 |
| LAT2 | 82.8% | 1.014 | 0.966 | 0.523 |
| CXADR | 88.5% | 1.005 | 0.966 | 0.504 |
| CTSH | 93.1% | 0.997 | 0.966 | 0.508 |
| CCL25 | 98.9% | 0.997 | 0.986 | 0.534 |
| BID | 87.4% | 1.001 | 0.987 | 0.540 |
| APLP1 | 100.0% | 1.002 | 0.987 | 0.503 |

Definition of abbreviations: AUC: area under the curve; FC: fold change; FDR: false discovery rate.

Differential protein levels between groups were calculated by linear models for array.

**Supplemental Table S3.** GO analysis for the proteomic signature associated with D_LCO_ through PLS-DA (STRING).

| GO category | False discovery rate | Strength | Observed protein count | Matching proteins in your network |
| --- | --- | --- | --- | --- |
| Positive regulation of cell differentiation | 8.40E-04 | 0.83 | 12 | HGF, IFNG, CLEC7A, METRNL, CALCA, PTN, CXCL9, ROR1, BAMBI, LILRB4, NPPC, PTK7 |
| Positive regulation of developmental process | 8.40E-04 | 0.75 | 14 | HGF, IFNG, CLEC7A, METRNL, CALCA, PTN, CXCL9, ROR1, CD40, BAMBI, LILRB4, NPPC, PTK7, PGF |
| Cell surface receptor signaling pathway | 4.10E-03 | 0.59 | 16 | HGF, IFNG, TFF2, CLEC4D, CXCL11, PTN, CXCL9, ROR1, CD40, BAMBI, NPPB, AREG, NPPC, PTK7, PGF, TNFRSF9 |
| Regulation of developmental process | 1.18E-02 | 0.53 | 16 | HGF, IFNG, CLEC7A, METRNL, CALCA, PTN, CXCL9, ROR1, CD40, BAMBI, NPPB, LILRB4, AREG, NPPC, PTK7, PGF |
| Regulation of interleukin-12 production | 1.33E-02 | 1.56 | 4 | IFNG, CLEC7A, CD40, TNFRSF9 |
| Immune response | 2.17E-02 | 0.63 | 12 | IFNG, LAMP3, CLEC4D, CLEC7A, CXCL11, CALCA, CXCL9, CD40, NPPB, CKAP4, LILRB4, CLEC5A |
| Signal transduction | 2.17E-02 | 0.38 | 21 | HGF, IFNG, TFF2, CLEC4D, CLEC7A, CXCL11, METRNL, CALCA, PTN, CXCL9, ROR1, CD40, BAMBI, NPPB, LILRB4, AREG, NPPC, PTK7, CLEC5A, PGF, TNFRSF9 |
| Regulation of cell differentiation | 2.17E-02 | 0.59 | 13 | HGF, IFNG, CLEC7A, METRNL, CALCA, PTN, CXCL9, ROR1, BAMBI, LILRB4, AREG, NPPC, PTK7 |
| Regulation of cell population proliferation | 2.22E-02 | 0.61 | 12 | IFNG, CLEC7A, CXCL11, PTN, CXCL9, EGFL7, CD40, BAMBI, AREG, NPPC, PGF, TNFRSF9 |
| Positive regulation of leukocyte activation | 2.76E-02 | 1.0 | 6 | IFNG, CLEC4D, CLEC7A, HAVCR1, CD40, LILRB4 |
| Positive regulation of immune system process | 2.81E-02 | 0.72 | 9 | IFNG, CLEC4D, CLEC7A, CALCA, PTN, HAVCR1, CD40, LILRB4, PGF |
| Inflammatory response | 2.81E-02 | 0.88 | 7 | IFNG, CLEC7A, CXCL11, CALCA, PTN, CXCL9, CD40 |
| Enzyme linked receptor protein signaling pathway | 2.81E-02 | 0.79 | 8 | HGF, PTN, ROR1, BAMBI, NPPB, AREG, NPPC, PGF |
| Cell chemotaxis | 2.81E-02 | 1.14 | 5 | HGF, CXCL11, CALCA, PTN, CXCL9 |
| Regulation of immune system process | 2.87E-02 | 0.61 | 11 | IFNG, CLEC4D, CLEC7A, SIGLEC7, CALCA, PTN, HAVCR1, NPDC1, CD40, LILRB4, PGF |
| Antimicrobial humoral immune response mediated by antimicrobial peptide | 2.99E-02 | 1.3 | 4 | CXCL11, CALCA, CXCL9, NPPB |
| Positive regulation of interleukin-12 production | 3.25E-02 | 1.62 | 3 | IFNG, CLEC7A, CD40 |
| Defense response | 3.38E-02 | 0.63 | 10 | IFNG, CLEC4D, CLEC7A, CXCL11, CALCA, PTN, TPSAB1, CXCL9, CD40, CLEC5A |
| Response to cytokine | 4.98E-02 | 0.66 | 9 | HGF, IFNG, LAMP3, TFF2, CXCL11, CALCA, CXCL9, CD40, TNFRSF9 |
| Positive regulation of multicellular organismal process | 4.10E-03 | 0.65 | 14 | HGF, IFNG, CLEC7A, CALCA, PTN, ROR1, CD40, BAMBI, NPPB, LILRB4, NPPC, PTK7, CLEC5A, PGF |
| Response to stimulus | 1.10E-02 | 0.29 | 28 | CCDC80, HGF, IFNG, LRP11, LAMP3, TFF2, CLEC4D, CLEC7A, CXCL11, METRNL, CALCA, PTN, TPSAB1, CXCL9, ROR1, CD40, BAMBI, NPPB, ENTPD6, IGFBPL1, CKAP4, LILRB4, AREG, NPPC, PTK7, CLEC5A, PGF, TNFRSF9 |
| Response to biotic stimulus | 2.17E-02 | 0.68 | 11 | CCDC80, IFNG, CLEC4D, CLEC7A, CXCL11, CALCA, CXCL9, CD40, NPPB, IGFBPL1, CLEC5A |
| Regulation of multicellular organismal process | 2.17E-02 | 0.47 | 17 | HGF, IFNG, TFF2, CLEC7A, CALCA, PTN, ROR1, CD40, BAMBI, NPPB, LILRB4, AREG, NPPC, PTK7, CLEC5A, PGF, TNFRSF9 |
| Cyclic-nucleotide-mediated signaling | 2.22E-02 | 1.2 | 5 | CXCL11, CALCA, CXCL9, NPPB, NPPC |
| Response to external stimulus | 2.49E-02 | 0.53 | 14 | CCDC80, HGF, IFNG, LRP11, CLEC4D, CLEC7A, CXCL11, CALCA, PTN, CXCL9, CD40, NPPB, CLEC5A, PGF |
| Response to organic substance | 2.49E-02 | 0.47 | 16 | HGF, IFNG, LAMP3, TFF2, CLEC7A, CXCL11, CALCA, PTN, CXCL9, CD40, BAMBI, AREG, NPPC, PTK7, PGF, TNFRSF9 |
| Regulation of ossification | 2.76E-02 | 1.15 | 5 | HGF, CALCA, PTN, AREG, NPPC |
| Positive regulation of biological process | 2.76E-02 | 0.32 | 23 | CCDC80, HGF, IFNG, LAMP3, CLEC4D, CLEC7A, CXCL11, METRNL, CALCA, PTN, HAVCR1, CXCL9, ROR1, EGFL7, CD40, BAMBI, NPPB, LILRB4, AREG, NPPC, PTK7, CLEC5A, PGF |
| Negative regulation of multicellular organismal process | 2.87E-02 | 0.66 | 10 | HGF, IFNG, TFF2, CALCA, PTN, NPPB, LILRB4, AREG, NPPC, TNFRSF9 |
| Cellular response to biotic stimulus | 2.87E-02 | 1.11 | 5 | CLEC7A, CXCL11, CXCL9, CD40, IGFBPL1 |
| Response to other organism | 2.95E-02 | 0.65 | 10 | CCDC80, IFNG, CLEC4D, CLEC7A, CXCL11, CALCA, CXCL9, CD40, NPPB, CLEC5A |
| Regulation of response to stimulus | 3.51E-02 | 0.39 | 18 | HGF, IFNG, CLEC4D, CLEC7A, METRNL, SIGLEC7, CALCA, PTN, ROR1, NPDC1, EGFL7, CD40, BAMBI, LILRB4, AREG, NPPC, PTK7, PGF |
| Ossification | 4.98E-02 | 1.02 | 5 | CHRDL2, VCAN, PTN, NPPC, CLEC5A |
| Regulation of anatomical structure morphogenesis | 4.98E-02 | 0.66 | 9 | HGF, PTN, CXCL9, ROR1, CD40, BAMBI, NPPB, PTK7, PGF |
| Positive regulation of cellular process | 4.98E-02 | 0.32 | 21 | CCDC80, HGF, IFNG, CLEC4D, CLEC7A, CXCL11, METRNL, CALCA, PTN, HAVCR1, CXCL9, ROR1, EGFL7, CD40, BAMBI, LILRB4, AREG, NPPC, PTK7, CLEC5A, PGF |

**Supplemental Table S4.** Pathway analysis for the proteomic signature associated with DLCO through PLS-DA (Reactome).

| Pathway name | p-value | Observed protein count | Matching proteins in your network | |
| --- | --- | --- | --- | --- |
| MET activates STAT3 | 9.01E-03 | 1 | HGF | |
| Immunoregulatory interactions between a Lymphoid and a non-Lymphoid cell | 1.20E-02 | 4 | NPDC1, CD40, SIGLEC7, LILRB4 | |
| Chemokine receptors bind chemokines | 1.29E-02 | 2 | CXCL9, CXCL11 | |
| MET interacts with TNS proteins | 1.50E-02 | 1 | HGF | |
| MET activates PTPN11 | 1.50E-02 | 1 | HGF | |
| MET Receptor Activation | 1.79E-02 | 1 | HGF | |
| MET activates PI3K/AKT signaling | 1.79E-02 | 1 | HGF | |
| Defective CHSY1 causes TPBS | 2.39E-02 | 1 | VCAN | |
| Defective CHST3 causes SEDCJD | 2.39E-02 | 1 | VCAN | |
| Defective CHST14 causes EDS. musculocontractural type | 2.39E-02 | 1 | VCAN | |
| VEGF ligand-receptor interactions | 2.39E-02 | 1 | PGF | |
| Signaling by Overexpressed Wild-Type EGFR in Cancer | 2.39E-02 | 1 | AREG | |
| Inhibition of Signaling by Overexpressed EGFR | 2.39E-02 | 1 | AREG | |
| VEGF binds to VEGFR leading to receptor dimerization | 2.39E-02 | 1 | PGF | |
| Phosphate bond hydrolysis by NTPDase proteins | 2.39E-02 | 1 | ENTPD6 | |
| EGFR interacts with phospholipase C-gamma | 2.68E-02 | 1 | AREG | |
| Constitutive Signaling by Aberrant PI3K in Cancer | 2.96E-02 | 2 | HGF, AREG | |
| MET receptor recycling | 2.97E-02 | 1 | HGF | |
| RUNX1 and FOXP3 control the development of regulatory T lymphocytes (Tregs) | 2.97E-02 | 1 | IFNG | |
| MET activates RAP1 and RAC1 | 3.27E-02 | 1 | HGF | |
| Dermatan sulfate biosynthesis | 3.27E-02 | 1 | VCAN | |
| Calcitonin-like ligand receptors | 3.27E-02 | 1 | CALCA | |
| MET activates RAS signaling | 3.56E-02 | 1 | | HGF |
| Physiological factors | 3.56E-02 | 1 | NPPC | |
| TNFR2 non-canonical NF-kB pathway | 3.80E-02 | 2 | CD40, TNFRSF9 | |
| WNT5A-dependent internalization of FZD2. FZD5 and ROR2 | 3.85E-02 | 1 | ROR1 | |
| GRB2 events in EGFR signaling | 4.14E-02 | 1 | AREG | |
| Regulation of IFNG signaling | 4.14E-02 | 1 | IFNG | |
| CS/DS degradation | 4.14E-02 | 1 | VCAN | |
| Post-translational protein phosphorylation | 4.14E-02 | 2 | CKAP4, VCAN | |
| SHC1 events in EGFR signaling | 4.43E-02 | 1 | AREG | |
| PI3K/AKT Signaling in Cancer | 4.79E-02 | 2 | HGF, AREG | |
| PI5P. PP2A and IER3 Regulate PI3K/AKT Signaling | 4.93E-02 | 2 | HGF | |

**Supplemental Table S5.** Drug-gene interaction for the proteomic signature associated with D_LCO_ levels (DGIdb 4.2.0).

| Target | Drug | Sources |
| --- | --- | --- |
| AREG | CETUXIMAB | CIViC  PharmGKB |
|  | PANITUMUMAB | CIViC  PharmGKB |
|  | CAPECITABINE | PharmGKB |
|  | IRINOTECAN | PharmGKB |
|  | CRIZOTINIB | CIViC |
| CALCA | EPTINEZUMAB | Chembl Interactions |
|  | PREDNISONE | NCI |
|  | DALFAMPRIDINE | TTD |
|  | AZD7009 | TTD |
|  | DRONEDARONE | TTD |
|  | DOFETILIDE | TTD |
|  | MINOXIDIL | TTD |
|  | AMIODARONE | TTD |
|  | AMIFAMPRIDINE | TTD |
|  | AZIMILIDE | TTD |
|  | CHEMBL1563246 | TTD |
|  | RANITIDINE | NCI |
|  | ANDOLAST | TTD |
|  | CIZOLIRTINE | TTD |
|  | PINACIDIL | TTD |
|  | BIMAKALIM | TTD |
|  | CHEMBL42661 | TTD |
|  | IBUTILIDE | TTD |
|  | BUDIODARONE | TTD |
|  | NATEGLINIDE | TTD |
|  | DIAZOXIDE | TTD |
| CAPG | VINCRISTINE | PharmGKB |
| CD40 | TENELIXIMAB | Chembl Interactions |
|  | DACETUZUMAB | Tdg Clinical Trial  Chembl  Interactions |
|  | ISCALIMAB | Guide To Pharmacology  TTD |
|  | PG-102 | Chembl Interactions |
|  | HYDROQUINONE | NCI |
|  | FLUDARABINE | NCI |
|  | RAVAGALIMAB | TTD |
|  | STREPTOZOCIN | NCI |
|  | APX-005M | TTD |
|  | BLESELUMAB | TTD |
|  | LUCATUMUMAB | Tdg Clinical Trial |
| EGFL7 | PARSATUZUMAB | TALC  Chembl Interactions  TTD |
| HGF | RILOTUMUMAB | TALC  MyCancerGenome  Tdg Clinical Trial  Chembl Interactions  Cancer Commons |
|  | FICLATUZUMAB | TALC  My Cancer Genome  Tdg Clinical Trial  Chembl Interactions  TTD |
|  | EPIGALOCATECHIN GALLATE | NCI |
|  | RESERPINE | NCI |
|  | STREPTOZOCIN | NCI |
|  | METHOTREXATE | NCI |
|  | ASPIRIN | NCI |
|  | MP-0250 | TTD |
|  | THALIDOMIDE | NCI |
|  | WORTMANNIN | NCI |
|  | IMATINIB MESYLATE | NCI |
|  | RESVERATROL | NCI |
| IFNG | FONTOLIZUMAB | Tdg Clinical Trial  Chembl Interactions |
|  | EMAPALUMAB | TTD |
|  | MELATONIN | NCI |
|  | CISPLATIN | NCI |
|  | IBUPROFEN | NCI |
|  | THEOPHYLLINE | NCI |
|  | PEFLOXACIN | NCI |
|  | TRASTUZUMAB | NCI |
|  | SURAMIN | NCI |
|  | PREDNISONE | NCI |
|  | BLEOMYCIN | NCI |
|  | AMIKACIN | NCI |
|  | CYCLOPHOSPHAMIDE | NCI |
|  | METHYLPREDNISOLONE | NCI |
|  | MELPHALAN | NCI |
|  | THROMBIN | NCI |
|  | AMITRIPTYLINE | NCI |
| NPPC | VASOPRESSIN | NCI |
|  | STAUROSPORINE | NCI |
|  | DIETHYLSTILBESTROL | NCI |
|  | HYDROCORTISONE | NCI |
|  | VOSORITIDE | TTD |
|  | PROGESTERONE | NCI |
|  | DEXAMETHASONE | NCI |
|  | ANDROSTENEDIONE | NCI |
|  | ETHER | NCI |
|  | TESTOSTERONE | NCI |
| PGF | AFLIBERCEPT | Chembl Interactions  TTD |
|  | CONBERCEPT | Chembl Interactions |
|  | TB-403 | Tdg Clinical Trial  TTD |
| PTK7 | COFETUZUMAB PELIDOTIN | TTD |
| ROR1 | CIRMTUZUMAB | TTD |
| TFF2 | CAMOSTAT | TTD |
|  | GABEXATE | TTD |
|  | APROTININ | TTD |
|  | NAFAMOSTAT | TTD |
| TNFRSF9 | UTOMILUMAB | Guide To Pharmacology  TTD |
|  | URELUMAB | Chembl Interactions  Guide To Pharmacology  TTD |
| TPSAB1 | GABEXATE | Guide To Pharmacology |
|  | CHEMBL256892 | Guide To Pharmacology |
|  | NAFAMOSTAT | Guide To Pharmacology |
|  | THROMBIN | NCI |
|  | REGRAMOSTIM | NCI |
|  | PENTAMIDINE | TTD |
| VCAN | CYCLOSPORINE | NCI |

ChEMBL: Ch European Molecular Biology Laboratory; CIViC: Clinical Interpretation of Variants in Cancer; NCI: National Cancer Institute; PharmGKB: the Pharmacogenomics Knowledge Base; TALC: Targeted Agents in Lung Cancer; TDG: The Druggable Genome; TTD: Therapeutic Target Database.

**SUPPLEMENTAL FIGURES**


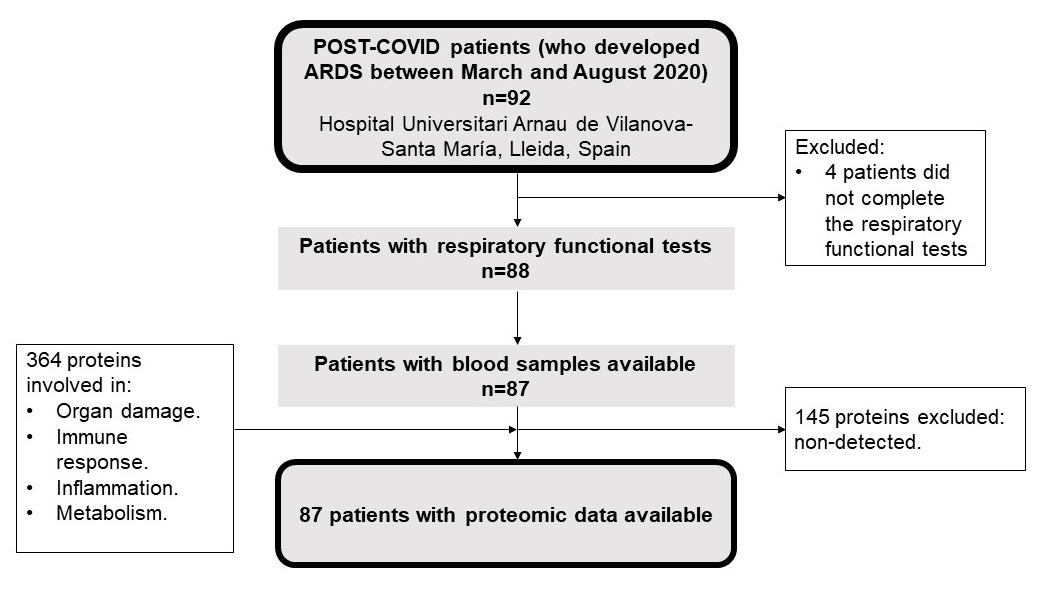


**Figure S1. Study flowchart.** Ninety-two patients with severe COVID-19 admitted to the Hospital Universitari Arnau de Vilanova-Santa María (Lleida, Spain) between March and August 2020 were enrolled if they fulfilled the following criteria: positive nasopharyngeal swab PCR or antigen test for SARS-CoV-2, aged over 18, developed ARDS based on the Berlin criteria during hospital stay and attended a “Post-COVID” evaluation 3 months after hospital discharge in the same hospital. The exclusion criteria for the “Post-COVID” evaluation included less than 1 year of life expectancy, transfer to another institution, treatment with palliative care, and inability to evaluate performance due to severe mental disability or incomplete pulmonary function evaluation. Finally, eighty-seven samples were available for proteome profiling. One hundred forty-five non-detected proteins were excluded from subsequent statistical analysis. The laboratory procedures were performed according to the standardized operating procedures of the Hospital Universitari Arnau de Vilanova-Santa María (Lleida, Spain).


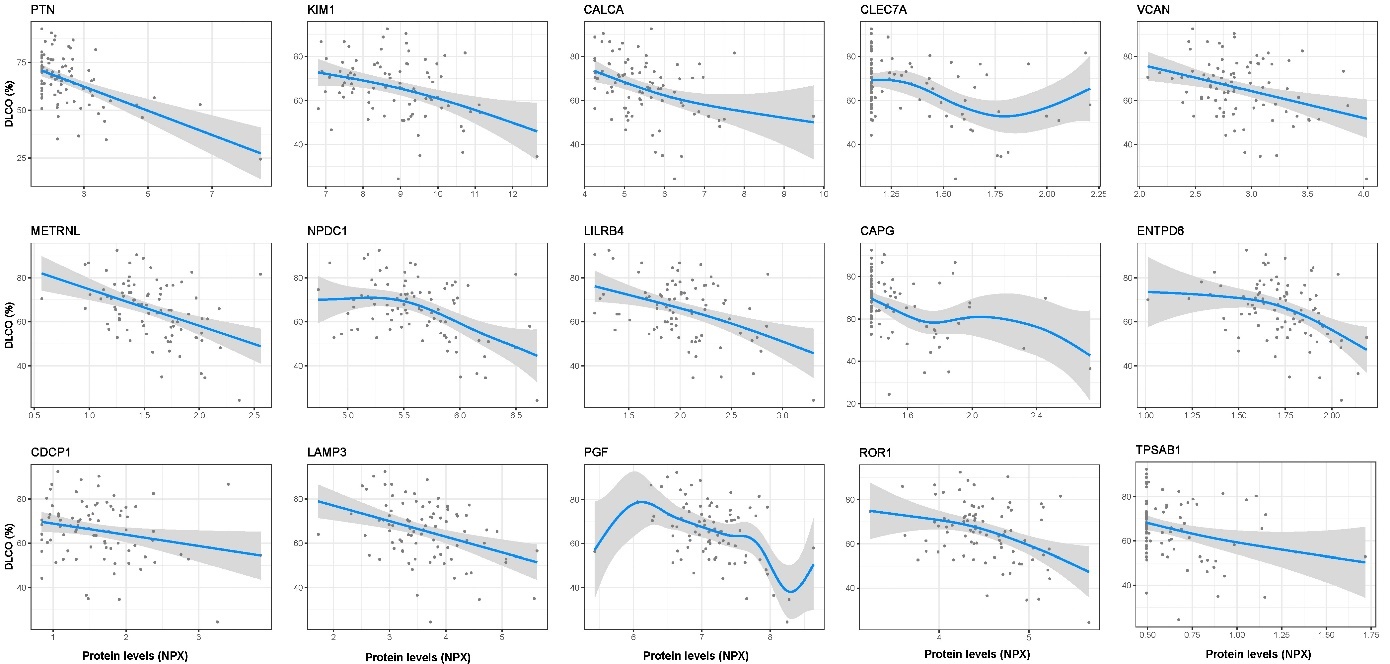


**Figure S2. Association between differentially detected proteins (FDR adjusted p-value <0.05) and D_LCO_ (without adjustment for confounding factors).** The dose–response relationship between the differentially detected proteins and D_LCO_ was analyzed using generalized additive models (GAMs) with penalized cubic regression splines.


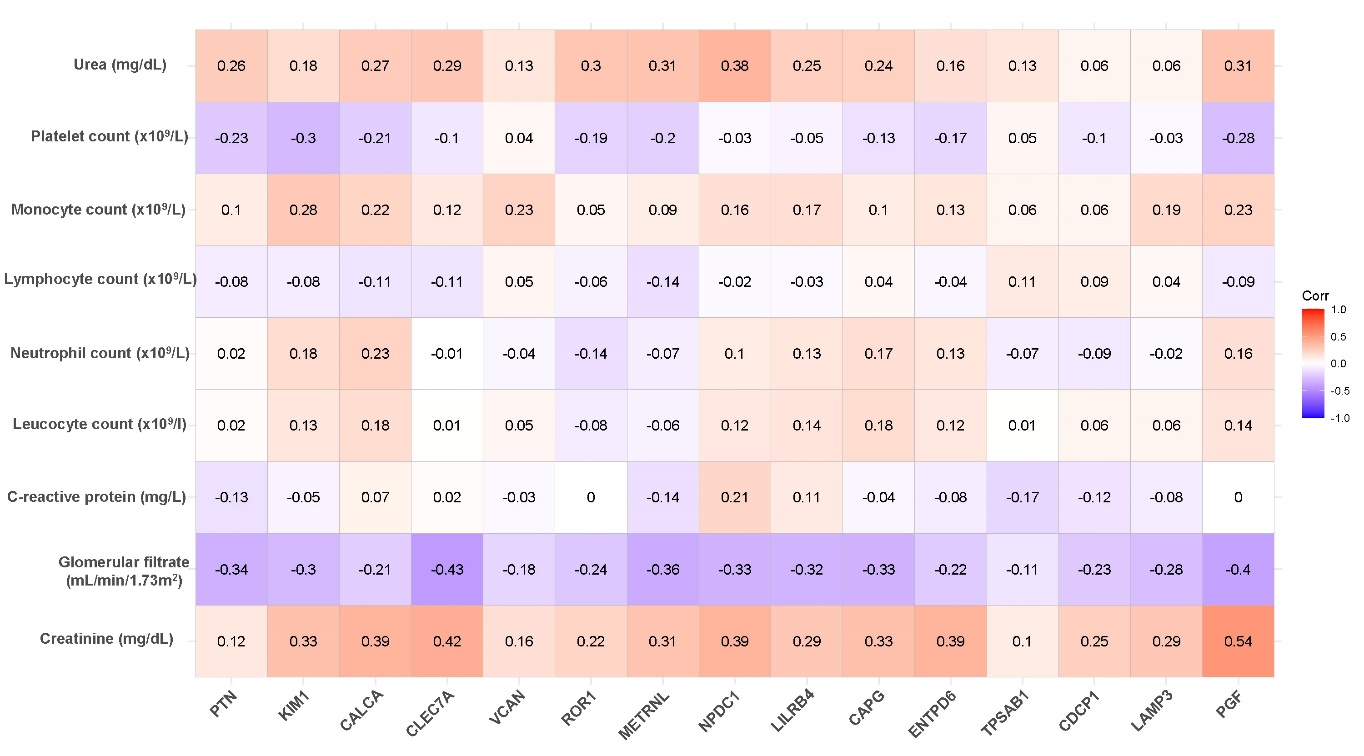


**Figure S3. Correlation between differentially detected proteins and laboratory parameters.** Spearman’s test was used to estimate the correlation between the variables.


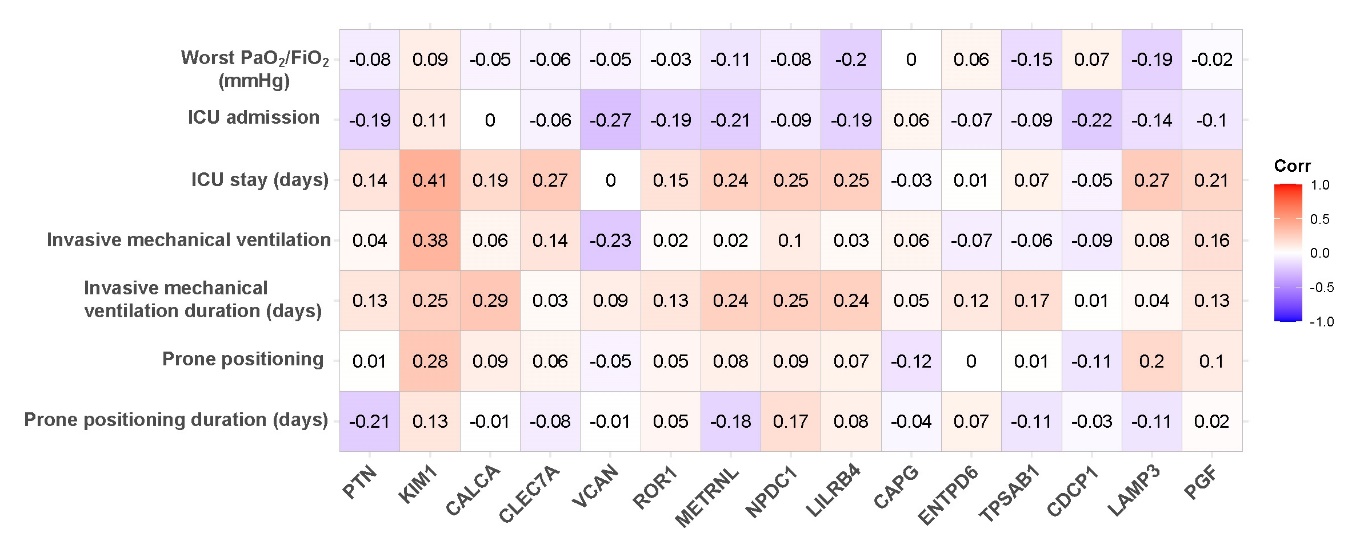


**Figure S4. Correlation between differentially detected proteins and clinical variables related to the severity of the acute phase.** Point-biserial correlation was used to estimate the correlation between dichotomous and continuous variables, and Spearman’s test was used to estimate the correlation between continuous variables.

**
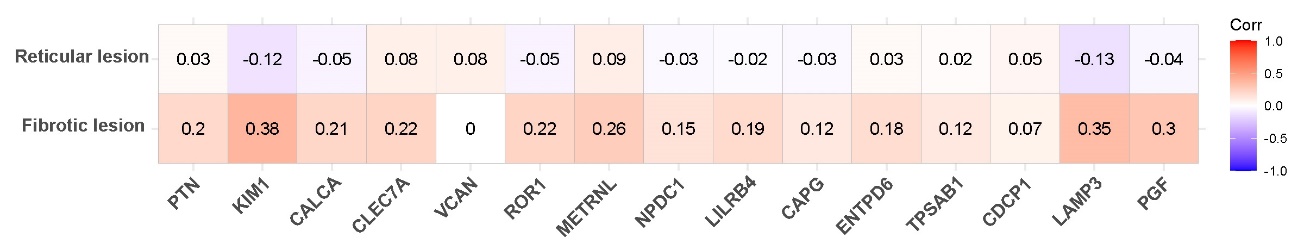
**

**Figure S5. Correlation between differentially detected proteins and reticular and fibrotic lesions.** Point-biserial correlation was used to calculate the correlation between categorical and continuous variables.

| **A**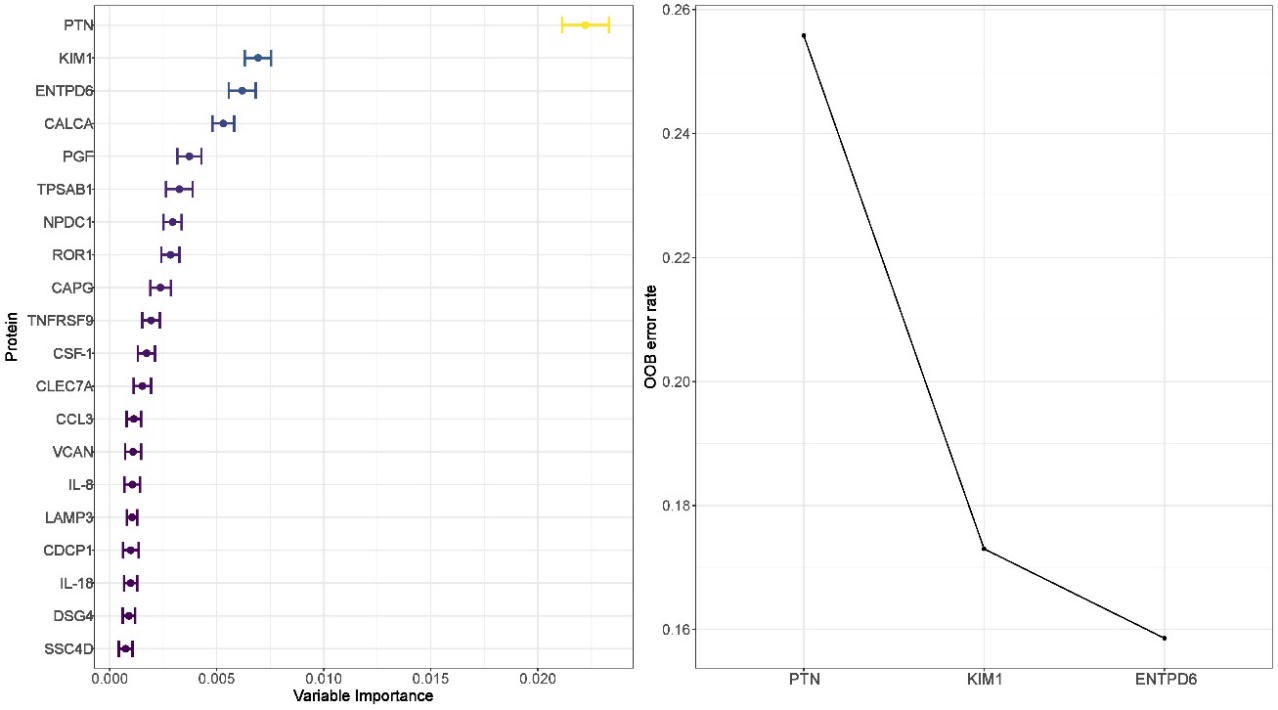 |
| --- |
| **B**  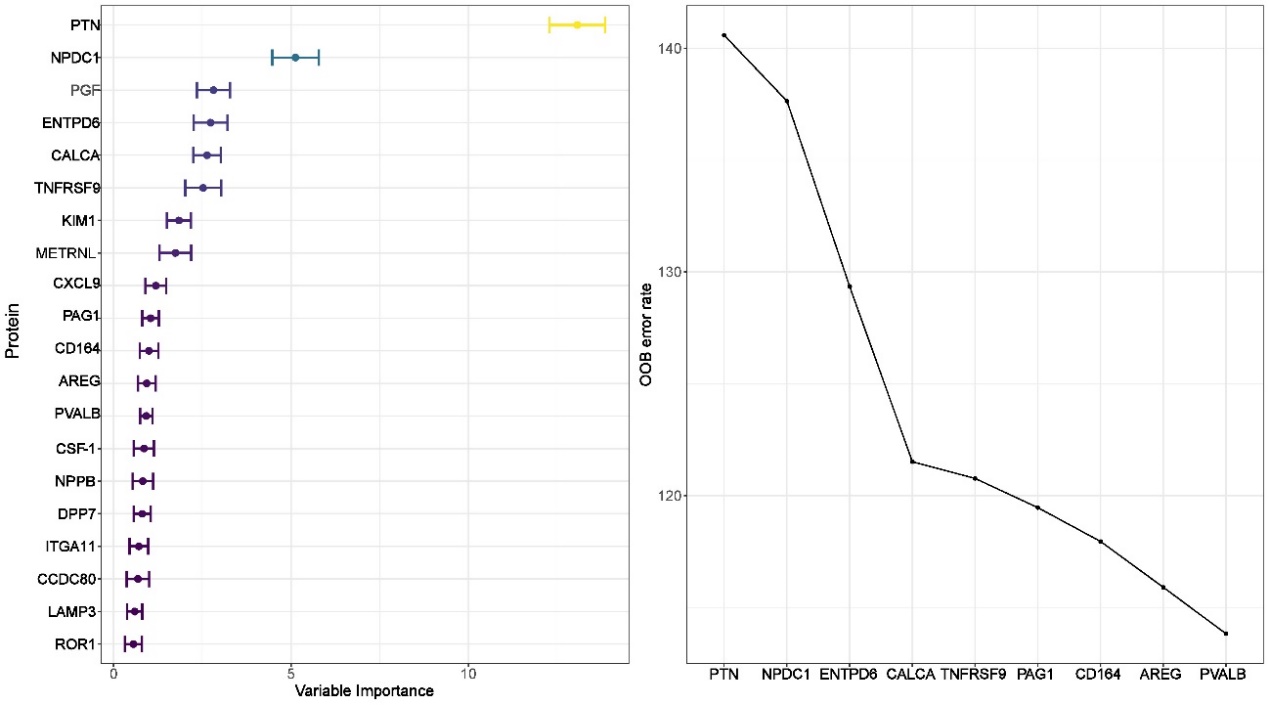 |

**Figure S6. Prediction model based on Random Forest.** (A) Prediction model for moderate to severe diffusion impairment (D_LCO_<60%). (B) Prediction model for D_LCO_. Random Forest algorithm was performed using 500 trees and an iteration number of 50, as main parameters. The importance of the contribution of each protein to the model is on the left. Variable importance was calculated as proposed in Genuer R et al. (Genuer R, Poggi JM, Tuleau-Malot C. Variable selection using random forests. Pattern Recognit Lett. 2010;31(14):2225-2236). The best combination of proteins selected by the algorithm to reduce the error is on the right.


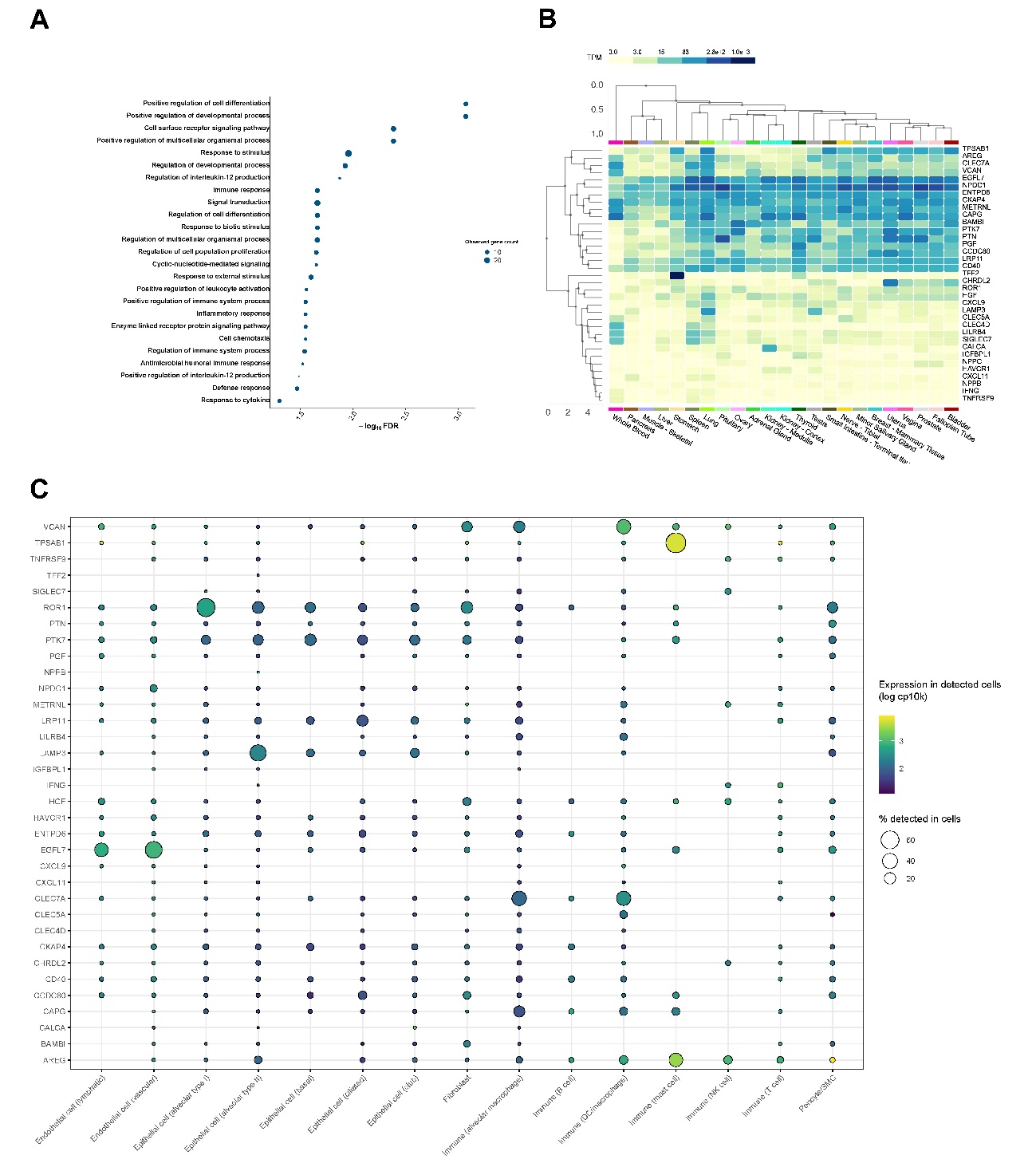


**Figure S7. Gene set enrichment analysis and cellular/tissue expression.** (A) Gene-set enrichment analysis considering proteomic signatures associated with D_LCO_ levels using STRING. The graph shows the FDR adjusted p-values of the top twenty-five GO terms. The size of each point is proportional to the number of proteins of the signature participating in the biological process. (B) Tissue and organ-enrichment analysis using Genotype-Tissue expression (GTEx). Hierarchical clustering shows tissues in the bottom and proteins on the right. (C) Cell enrichment analysis based on single-cell RNA-seq for the proteome signature using GTEx. Each column represents a cell type and each row represents a protein. The size of the point shows the number of cells where the gene expression has been detected and the color represents the expression level. The GTEx Project was supported by the Common Fund of the Office of the Director of the National Institutes of Health and by the NCI, NHGRI, NHLBI, NIDA, NIMH, and NINDS.


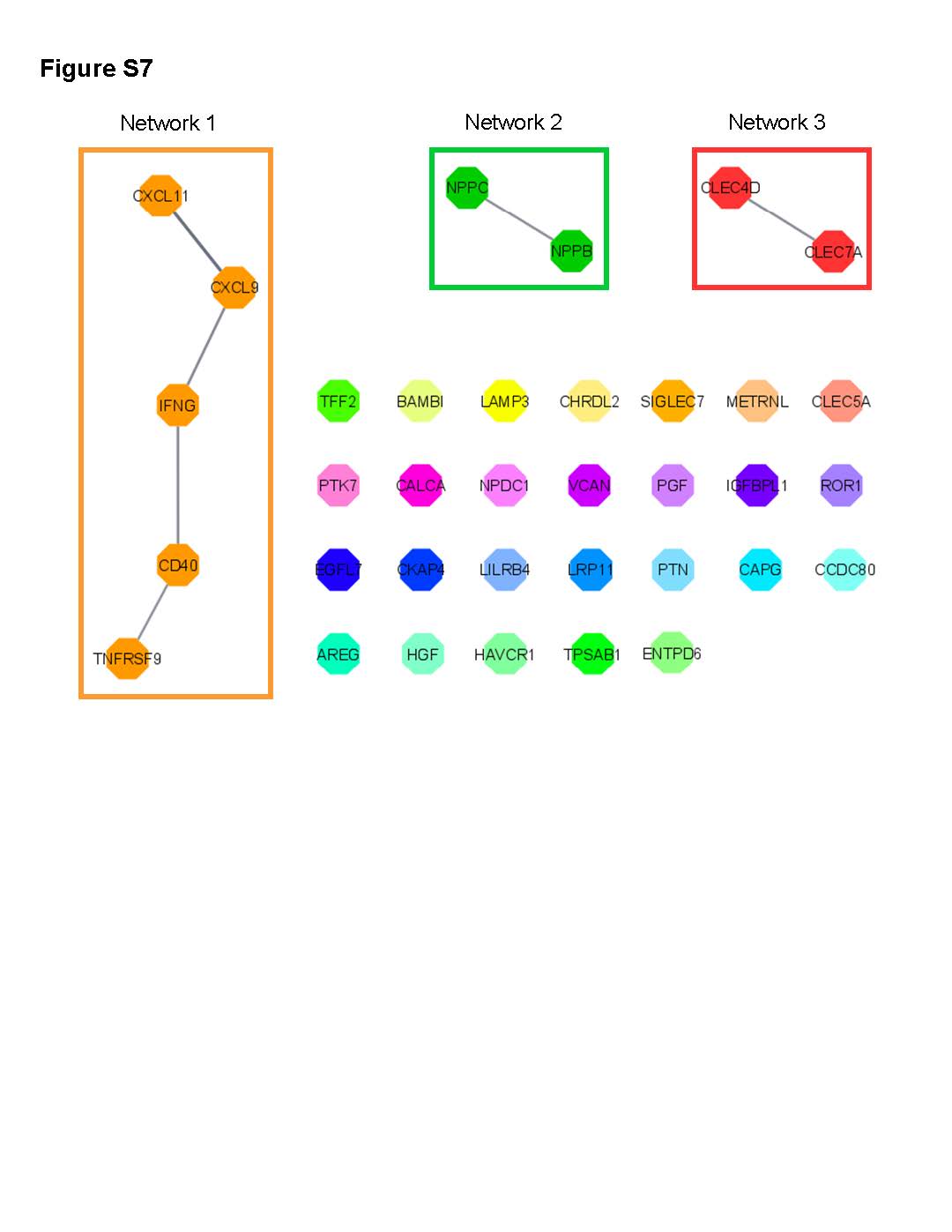


**Figure S8. PPI network of the protein signature associated with D_LCO_.** The PPI network was constructed using Cytoscape 3.8.2.
